# Emerging importance of holobionts in evolution and in probiotics

**DOI:** 10.1186/1757-4749-5-12

**Published:** 2013-05-22

**Authors:** Yadvir Singh, Javed Ahmad, Javed Musarrat, Nasreen Z Ehtesham, Seyed E Hasnain

**Affiliations:** 1 Kusuma School of Biological Sciences, Indian Institute of Technology, Hauz Khas, New Delhi 110016, India; 2 Department of Zoology, College of Science, King Saud University, Riyadh, Saudi Arabia; 3 National Institute of Pathology, Safdarjang Hospital, New Delhi, India; 4 Dr Reddy’s Institute of Life Sciences, University of Hyderabad Campus, Professor CR Rao Road, Hyderabad 500046, India

**Keywords:** Evolution, Symbiont, Hologenome, Holobiont, Probiotics

## Abstract

The existence of microbe free animals or plants in nature is virtually impossible as they and plants have a certain degree of symbiotic association with microbes. This symbiotic association leads to the formation of holobiont (host and its symbionts). This mutual coexistence is not merely at the physical or chemical level but also at the genetic level leading to the emergence of the concept of hologenome (gene pool of host and its associated symbionts). The abundance of symbionts with the associated gene diversity contributes to the fitness of the holobiont under varying environmental conditions. The hologenome theory of evolution considers the dynamic holobiont as a single unit for natural selection and provides a more accommodating view of evolution blending Darwinism and Lamarkism. Additionally, holobionts are providing scientific basis to our understanding of the growing importance of probiotics in human health and in disease management.

## Introduction

**Figure 1:**
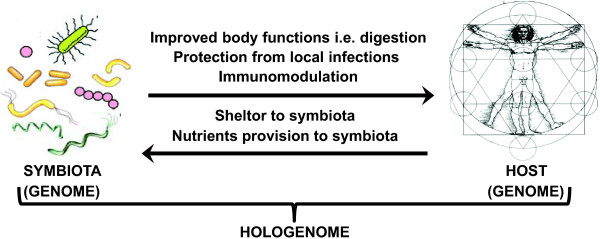
**The concept of hologenome.** Hologenome consist of genome of holobiont which includes host and its associated symbiota having a mutual beneficial relationship. Symbiota not only protects the host from pathogens but also decreases immune disorders by immunomodulation. While host provides shelter and nutrients to the symbiota, symbiota in turn also improve various body functions such as digestion to provide essential nutrients to the host.

During the course of evolution, multicellular living forms emerged from unicellular life; the latter not only predominates the multicellular life quantitatively but also has a close association with it. There are many types of associations that have developed during evolution ranging from mutualism to parasitism. Such associations always affect the life of multicellular hosts in the short term from birth to death, with implications on their survival in the environment, and in the long term as a phenotypic unit for natural selection. Such relationships lead to formation of a holobiont [1,2] that includes host and its associated microbiota or symbionts (Figure 1). Although the word “Holobiont” was coined by Lynn Margulis in 1991, to signify symbiosis between individual organisms or the bionts [1], the concept was highlighted in 2002 and later during the study of corals, and their associated symbionts, as coral holobionts [2]. Symbionts can be divided into two categories, endosymbionts and exosymbionts which refer to symbionts living inside or outside of the host cells, respectively. Such associations result in a hologenome comprising of genetic information of both host and the associated microbiota [3]. Hologenome includes the static genome of the host alongwith dynamic genome of the symbiota. The dynamism of symbiota’s genome provides the competence to holobiont to adapt and survive in different environmental conditions. The hologenome theory of evolution considers the alliance of holobiont with its hologenome as a selection unit for evolution to act upon [3]. Further hologenome theory of evolution can be helpful in comprehending the emerging constructive role of probiotics in human health.

## Hologenome theory and evolution

Natural selection acts at multi-levels i.e. at the genetic level, individual level or community level. Recent reports suggest that natural selection also operates at the level of holobiont. The hologenome theory of evolution, proposed by Zilber-Rosenberg and Rosenberg [4], is based on the following assumptions:

1) There is abundance and diversity in microbial population housed by all animals and plants.

2) The microbial symbionts influence their holobiont fitness and *vice versa*.

3) Modifications in the host genome or microbiota genome result in variations in the hologenome.

4) Transmission of hologemone variations can occur with fidelity.

This theory came into existence based on observation on *Vibrio shiloi* infection in corals. With time corals became resistant to infection despite the fact that they lack an advanced immune system [5]. In case of coral holobionts, microbiota help the corals to adapt to changing environmental conditions [6]. For example in the bleaching process, when due to increase in sea water temperature, corals start to lose their symbiotic zooxanthallae. These symbionts act as a source of carbon and molecular oxygen. According to adaptive hypothesis of coral bleaching [7], the expulsion of algae paves way for the infection of temperature resistant zooxanthallae, which led to a more favorable symbiosis. This led to the presentation of coral probiotic hypothesis [8]. Thus, the existence of dynamic relationship between symbionts and corals under different environmental conditions led to the selection of most advantageous coral holobiont in the background of prevailing conditions.

### Abundance and diversity in microbial population housed by all animals and plants

Microbes are the most abundant organisms on this earth. Surface of animals and plants is always in physical contact with water, soil or air which in turn contains microbes as a function of a unit area. Previously known culture dependent isolation techniques for these host associated bacteria underestimated their diversity. Recently developed culture independent molecular biology techniques like 16S rRNA sequence analysis and high throughput sequencing have suggested a fresh estimate of the number of microbial species associated with the hosts. For example, an average human body, made up of 10 trillion cells, is now estimated to harbor 100 trillion cells representing distinct microbial species in the gut alone. According to these figures, the number of unique bacterial genes alone in the human gut becomes ~150 times more than the number of human genes [9]. Other human body systems like skin, urogenital system and respiratory system also harbors microbes with a great abundance and diversity. The potential for variations in hologenome is thus increased as a function of the number of unique symbiont genomes.

### The microbial symbionts influence their holobiont fitness and *vice versa*

Genetic diversity of microbiota can help the holobiont to increase the efficiency of adaptation to the variations in environment. While the host provides a sheltered and nutrient rich environment for survival and growth of microbiota, in extreme cases like in absolute mutualism, survival of both host and the symbiont is completely dependent on each other. Extreme symbionts such as mitochondria and chloroplasts can directly influence the survival of their host as mutations affecting their function lead to various diseases in the host. Symbiota also help in adaptation in varying environmental conditions. For example in bovine rumen symbiosis, microbiota help the host to get energy and nutrients from complex plant material by providing various bacterial enzymes that are otherwise not synthesized by the host. In case of human gut holobiont, research on germ free animals indicated that the normal microbiota help in development and functioning of normal immune system [10-12], angiogenesis [13], regulation of fat accumulation [14] along with food breakdown and biotransformation. Alteration in metabolism modulated by progressive changes in microbiota composition in humans during the course of pregnancy has been found to be supportive for the growth of fetus [15]. Recently it was found that caesarean section delivery of infants may make them more prone to obesity as they did not acquire the normal microbiota which would have been acquired during normal vaginal delivery [16].

Normal microbiota also competes for space with the pathogenic microbes to provide protection against infectious disease in the host. For example in human, microbiota on mucosal surface resist infection by mucosal pathogens through antibacterial release and binding to adhesion sites [17]. Interestingly, it has been shown that immune response to symbiota *via* IgM differs from its reaction to pathogens [18]. It was further observed that human skin symbiota composition affects the attractiveness to mosquitoes by producing different human odour volatiles as their metabolites [19]. This may alter their susceptibility to malaria as malarial parasites are transmitted by these mosquitoes.

During the course of evolution, symbiosis between reteroviruses and primitive egg laying mammals is believed to have led to the development of placental mammals through genetic integration of reteroviral genes in the host [20]. Other examples include those where the coral symbiota help in nitrogen fixation [21], nutrients assimilation [22] and prevention of infections by antibiotic production [23-25].

Termites with their symbionts in the gut represent examples where they not only help in nutrient assimilation but also affect the social behavior [26]. This is evident from study of diet induced mating preference in Drosophila due to symbionts [27] that alter the levels of cuticular hydrocarbon sex pheromones to induce a mating preference. Alongwith geographic separation and slower changes in host genomes, such mating preference will lead to the evolution of species [28,29]. In some rare cases, like in coffee beetle, a gene important for beetle survival has been found to have bacterial origin. This gene might have been acquired during evolution by the beetle from the bacteria through nonsexual horizontal gene transfer [30]. In case of pea aphid *(Acyrthosiphon pisum)*, a symbiont bacterial species *(Hamiltonella defensa)* acts as an anti-parasite defender by killing the developing parasitic wasp larvae, thus enhancing the survival of the host [31].

Symbionts have also been found to play important role in organogenesis in host. For example, the symbiont *Vibrio fischeri* helps in development of squid light organ of Hawaiian bobtail squid *Euprymna scolopes*[32,33]. Both can grow independently but in the absence of symbiont there will be no light organ formation. This observation points to the existence of interspecies signaling.

### Modifications in the host genome or microbiota genome result in variations in the hologenome

Inequality of traits, termed as variations, synthesize the basic substrate for evolution. Variations in host genome can be acquired during sexual reproduction, recombination, rearrangement of chromosomes, and due to genetic and epigenetic mutations. In haploid bacteria recombination occurs through transduction, DNA transformation and conjugation. In symbionts, besides recombination and the methods mentioned above, there are three other processes by which variations can be acquired. These include: microbial proliferation, acquisition of exotic strains, and horizontal gene transfer. Unique to the hologenome theory of evolution, these processes can occur rapidly depending on environmental need and are important factors in the adaptation and evolution of animals and plants [4].

#### Microbial proliferation

The proliferation of microbes is the most accelerated mode of generating variation in holobionts. It includes changes in the relative abundance of the various types of associated symbionts that can occur as a consequence of changing environmental factors. Relative increase in the number of a specific microbe is analogous to gene amplification. Considering the abundance and diversity of microbiota, this process becomes a potent mechanism for adaptation in changing environment. Diet induced change in gut microbiota is the most studied example [34,35].

#### Acquisition of exotic strains

During the life time, animals and plants come in contact with billions of microbes. There are chances when a particular bacterium finds an appropriate niche for establishment in the host leading to symbiosis. Certain environmental conditions can render a new symbiont more abundant, resulting in alteration in the holobionts phenotype. Unlike microbial amplification, acquiring new symbionts from the environment can result in import of entirely novel genes into the hologenome. For example, the administration of probiotics restores the lost beneficial bacterial strains during antibiotic therapy in various infectious and noninfectious gastrointestinal disorders [36]. In case of whitefly, the symbiont *Rickettsia* is initially acquired through feeding on the infected plant resulting in a plant mediated horizontal transmission in the insect [37].

#### Horizontal gene transfer

Horizontal gene transfer from exotic to resident microbe is an additional robust method for generating variability in symbionts. An example is the transfer of porphyranases, agarases and associated protein coding genes from a marine member of the bacteroidetes to the human gut bacterium *Bacteroide splebeius* in the Japanese population [38]. This process is mostly mediated by mobile genetic elements like transposons, plasmids, bacteriophages and genomic islands. Interestingly, genomic islands are present in pathogens as pathogenicity islands [39], as well as in beneficial symbionts as symbiosis islands [40] and are responsible for mediating bacteria-host interactions. Genetic information exchange between pathogens and symbionts may also be brought by horizontal gene transfer [41,42].

### Transmission of hologemone variations can occur with fidelity

Trans-generational transmission of host genetic information in an error proof manner has been well characterized. In recent years, similar type of information for symbionts has started to emerge [43]. There are two different types of modes of transmission: horizontal (from environment) and vertical (from parents/mother). However a mixture of both modes is also prevalent in nature. There are several examples of direct mode of transmission or trans-ovarian mode. Cytoplasmic inheritance of mitochondria and chloroplast (presumed as extreme symbionts) occurs when a eukaryotic cell divides. In the aphid–Buchnera symbiosis, bacteria are intracellularly present in specialized host cells called bacteriocytes and are transferred to next generation by trans-ovarian transmission [44].

Other than trans-ovarian transmission, there is transmission through direct contact in several cases. For example, in humans, initial symbiont population is acquired during passage through birth canal and later by close physical contact with family. It was observed that microbiota similarity in a human population was more within family than between families in a particular area [45,46].

## Role of microbiota in human health: importance of probiotics

**Figure 2:**
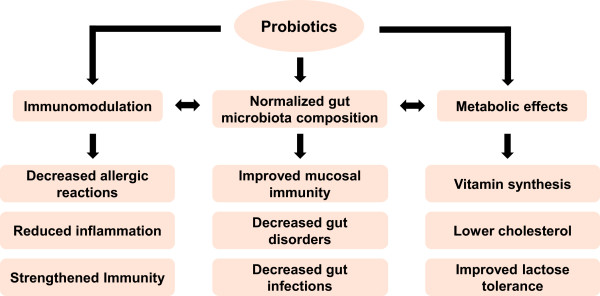
**Importance of gut probiotics in human health.** The probiotics play an important role in maintaining normal microbiota composition, metabolism and immunity. All these functions of probiotics are correlated with each other. Normal microflora decrease the gut infections and disorders alongwith strengthening the mucosal immunity. Probiotics as normal microflora help in synthesis of vitamins e.g. vitamin K, metabolism of lactose and lowers the level of cholesterol. Probiotics enhance the systematic immunity which further reduces the chances of allergy and inflammation by immunomodulation.

**Figure 3:**
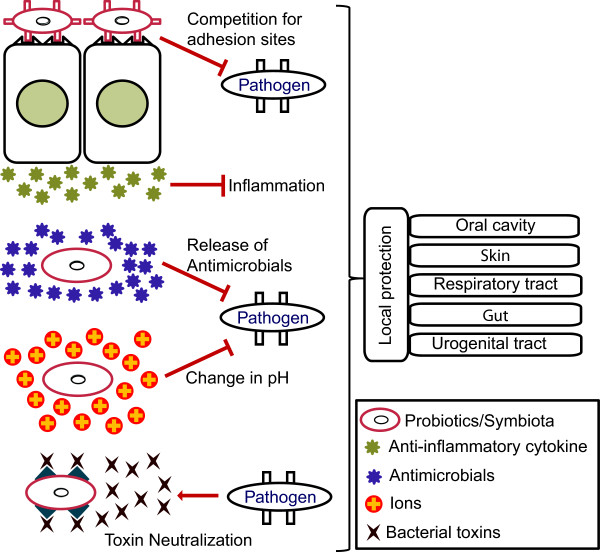
**Probiotics impact mucosal/surface immunity.** Probiotics can provide local or mucosal immunity and protection from many pathogens in a diversified manner. They can affect the adhesion of different pathogens to host cell surface by competitive exclusion. The binding of probiotics can induce the host cells to secrete anti-inflammatory cytokines which will decrease the inflammation at the tissue surface. Probiotics can also secrete antimicrobials to inhibit the growth of pathogens. The metabolites of probiotics like lactic acid from lactobacillus bacterium can result in alteration of pH in lumen or at surface, which can ultimately inhibit the growth of certain pH sensitive pathogens. Probiotics can also bind to toxin released by pathogens resulting in neutralization of the toxins. Similar types of protection can be provided by symbiota associated with other organs i.e. oral cavity, skin, respiratory tract and urogenital tract.

**Figure 4:**
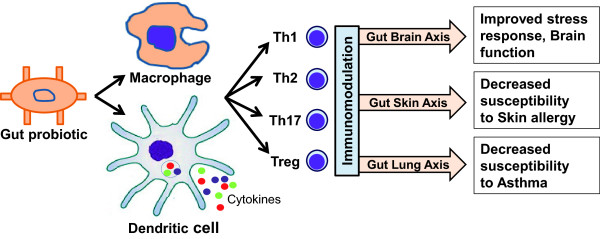
**Role of probiotics in systemic immunity.** Probiotics can improve the generalized immunity by immunomodulation. In immunomodulation, gut probiotics interact with antigen presenting cells i.e. macrophages and dendritic cells (DC). These APC then affect the T cells population (mainly regulatory cells) to induce systemic immunomodulation. This immunomodulation through gut-brain axis, gut-lung axis and gut-brain-skin/gut-skin axis improves the function of brain, lungs and skin respectively, by reducing the susceptibility to various stresses and allergies.

According to World Health Organization/Food and Agricultural Organization (2001), probiotics are defined as live microbial species which when ingested in sufficient amount render beneficial effects on human and animal health [47]. The gut symbiota (probiotics) play an important role in maintaining normal microbial composition, metabolism and immunity of gut (Figure 2). All these functions of probiotics are correlated to each other. Probiotics not only provide protection against pathogens at local or mucosal surfaces but also play an important role in the development of systemic immunity through immunomodulation. Local protection is provided by various means. Probiotics compete for binding to adhesion sites at host cell surface that are also required by a pathogen. Further, probiotics can release many types of antibacterial which can either kill or inhibit the growth of pathogens. Change in the milieu i.e. alteration in pH through secretion is another way to inhibit the growth of pathogens by probiotics. Binding of probiotics to host cell receptors can induce the host cells to secrete various anti-inflammatory compounds which results in amelioration of inflammatory response/tissue damage [48]. Probiotics can also bind to bacterial toxins resulting in toxin neutralization [49]. Similar type of protection can be provided by symbiota associated with other organs i.e. oral cavity, skin, respiratory tract and urogenital tract (Figure 3). Probiotics also improve immunity through immunomodulation. In immunomodulation, gut probiotics interact with antigen presenting cells i.e. macrophages and dendritic cells. Upon interaction with probiotics these cells secrete certain cytokines which regulate the function of certain regulatory T cells, resulting in immunomodulation [50]. This immunomodulation (Figure 4) results in an effective immune system by decreasing the susceptibility to various inflammations and allergies through various gut-organ axises i.e. gut-brain axis, gut lung axis and gut-skin axis [51-54].

Probiotics have already been exploited either to treat or to prevent a number of gut health disorders such as irritating bowel syndrome [55], hypersensitivity including food allergies [56], hypercholesterolemia [57], gastritis, gut infection [58], parasitic infestation [59] and even certain forms of cancers including colorectal cancer [60]. Microbiota as probiotics also hold potential for use in oral health to prevent and treat the oral infections, dental plaque-related diseases, periodontal diseases and halitosis [61]. Intestinal microbiota also modulate the immune system (Figure 4), thus decreasing the chances of getting allergies of skin like eczema and of respiratory system like asthma [62]. Urogenital microbiota also help to reduce the urogenital infection in woman by preventing pathogen adhesion and secretion of antibacterial compounds [63]. Results from animal studies have shown the important role of microbiota in formation of microbiome-gut-brain axis, which can affect brain and behavior [64]. Probiotics can also modulate brain functions including stress response in humans [65]. Symbiota associated with other organs also hold similar potential to treat or prevent human health disorders linked to those organs. Given the important role of probiotics on the host metabolism and function, one can argue whether it is appropriate to assign definitive functions to host genes in the absence of knowledge about cross-talks between host and the symbiont genes.

## Genetically engineered probiotics: potential of recombinant probiotics

Recombinant DNA technology makes it possible to increase the prophylactic and therapeutic efficacy of existing probiotic strains as well as to create completely new probiotic strains. Through improvement of stress tolerance during pharmaceutical formulation and in *in vivo* conditions, technological robustness and clinical efficacy of a probiotic formulation can be improved. This task can be accomplished with the help of pathobiotechnology, in which pathogenic bacteria are exploited for their better stress response, host interaction and survival within host for constructive use in biotechnology [66]. For example, a better stress tolerance of the bacteriocin producing probiotic bacterium *Lactobacillus salivarius* UCC118 was observed when a betaine uptake system gene involved in osmotic stress tolerance was cloned from *Listeria monocytogenes* into the probiotic strain [67]. Genetically modified probiotics can also be used in replacement therapies in which a pathogenic microbe is replaced by its harmless and robust competitor in the same ecological niche. For example, the cariogenic wild type bacterium *Streptococcus mutans* was replaced by a mutant lacking lactic acid producing gene responsible for dental caries [68].

Genetic engineering of probiotics has also led to the emergence of concept of designer probiotics. These probiotics are genetically engineered to express the mimics of host cell receptors for pathogenic microbes on surface [49]. Such probiotics can be used in effective toxin sequestration, neutralizing antibody production, and detoxification [69]. Recently scientists have also started to exploit the role of probiotics as vaccine, immune cell mediators and delivery systems. The recent report of therapeutic and prophylactic molecules delivery by lactic acid bacteria [70] promises to render these modified probiotics for use in anti-inflammatory, anti-tumor and anti-viral therapies.

Genetically modified symbiota in other species can also have medical applications. For example the recent engineering of a mosquito symbiotic bacteria to secrete anti-*Plasmodium* effector proteins making them resistant to malarial infection [71] represent a new strategy for intervention against malaria. Genetically engineered symbiota holds the potential to enhance the production performance of various animals and plants associated with human society.

## Conclusion and epilogue

The hologemone concept evaluated the combination of host and symbiota genomes as a single unit for natural selection by environmental forces leading to the evolution of holobiont. Microbial diversity has been found to affect fitness in terms of adaptation and survival of the host in various environmental conditions. Change in coral associated flora during bleaching process, diet induced changes in gut microbiota population diversity, and altered mating preference in Drosophila and Vibrio associated organ morphogenesis in squid are some examples. Early evolutionary theories fail to consider the role of microbial diversity in evolution of animal or plant host. These evolutionary theories were mainly influenced by Darwin, Lamark, Weismann and Mendel. Darwin’s Natural selection selects only those phenotypes which are beneficial to host in terms of struggle for existence and survival of fittest. Lamark’s use or disuse of characteristics has an effect on their long term development and traits acquired during lifetime can be transferred to the next generation. Weismaan proposed that germ cell inheritance occurs by germ cells which are not affected by acquired traits of somatic cells. Mendelian genetics considered random mutations as the source for variations leading to evolutions. All these theories were unable to explain all types of inheritance observed in nature. Thus inheritance of DNA sequence independent changes in terms of epigenetic inheritance lead to emergence of neo-Lamarkism which was able to cross Weismaan barrier of germ cell inheritance of acquired traits. The hologenome theory of evolution signifies the amalgam of Darwinism and Lamarckism [72]. It considered the highly dynamic system of holobiont as a single unit for natural selection leading to evolution as well as inheritance of acquired characters in the form of change in symbiotic population modulated by environmental forces.

Darwin’s four postulates hold true for holobiont. 1) Each holobiont differs from the other holobionts in terms of diversity and abundance of symbionts associated with host. 2) The diversity and abundance are directly related to the potential for variation in hologenome and transmission of hologemone variations can occur with fidelity. 3) The microbial symbionts affect their holobiont fitness, which can depend upon diversity and relative abundance of symbionts. This fitness can affect the survival and reproduction in case of holobiont. 4) Holobionts with high potential of fitness are most likely to survive for reproduction.

Due to dynamic nature of holobiont, there can be changes in relative diversity of symbionts as directed by the environmental forces. The use and disuse of a particular character in terms of symbiont phenotype over time justify Lamark’s first principle of use and disuse. Lamark’s second principle of inheritance of acquired characters also holds true in case of holobiont as variations in holobionts can be transmitted to next generation with fidelity.

Hologenome theory of evolution helps better understand fitness potential of the holobiont. It also provides a tool to alter the hologenome to increase the fitness by altering the dynamic symbiont population. As it is difficult to alter the host genome, an alteration of the hologenome through symbiont’s genome variations can render better host survival under harsh conditions.

Concepts of pathobiotechnology, designer probiotics and replacement therapy have highlighted the potential of modified probiotics for human health. The four assumptions of the hologenome theory of evolution [4] will not only help evaluate the constructive role of the natural probiotics in human health but also predict the outcome of interactions of genetically modified probiotics with the human holobiont in the long term. Generation of modified probiotics also requires an efficient biological containment system to prevent the potential spread of the modified genetic trait across the natural symbiota.

Thus a deep understanding of hologenome theory will greatly aid in harnessing the therapeutic potential of microbiota in human health from the clinical perspective.

## Competing interests

The authors declare that they have no competing interests.

## Authors’ contributions

YS reviewed literature, collected data, outlined and drafted the manuscript; JA collected data and participated in writing the manuscript; JM collected data and outlined the final draft; NZE reviewed the draft and assisted in preparing the final version; and SEH conceived the article, reviewed literature, and wrote the article in its final format. All authors read and approved the final manuscript.
